# Serial Change in Cervical Length for the Prediction of Emergency Cesarean Section in Placenta Previa

**DOI:** 10.1371/journal.pone.0149036

**Published:** 2016-02-10

**Authors:** Jae Eun Shin, Jong Chul Shin, Young Lee, Sa Jin Kim

**Affiliations:** Department of Obstetrics and Gynecology, College of Medicine, The Catholic University of Korea, Seoul, Republic of Korea; Hospital de Especialidades del Niño y la Mujer de Queretaro, MEXICO

## Abstract

**Purpose:**

To evaluate whether serial change in cervical length (CL) over time can be a predictor for emergency cesarean section (CS) in patients with placenta previa.

**Methods:**

This was a retrospective cohort study of patients with placenta previa between January 2010 and November 2014. All women were offered serial measurement of CL by transvaginal ultrasound at 19 to 23 weeks (CL1), 24 to 28 weeks (CL2), 29 to 31 weeks (CL3), and 32 to 34 weeks (CL4). We compared clinical characteristics, serial change in CL, and outcomes between the emergency CS group (case group) and elective CS group (control group). The predictive value of change in CL for emergency CS was evaluated.

**Results:**

A total of 93 women were evaluated; 31 had emergency CS due to massive vaginal bleeding. CL tended to decrease with advancing gestational age in each group. Until 29–31 weeks, CL showed no significant differences between the two groups, but after that, CL in the emergency CS group decreased abruptly, even though CL in the elective CS group continued to gradually decrease. On multivariate analysis to determine risk factors, only admissions for bleeding (odds ratio, 34.710; 95% CI, 5.239–229.973) and change in CL (odds ratio, 3.522; 95% CI, 1.210–10.253) were significantly associated with emergency CS. Analysis of the receiver operating characteristic curve showed that change in CL could be the predictor of emergency CS (area under the curve 0.734, p < 0.001), with optimal cutoff for predicting emergency cesarean delivery of 6.0 mm.

**Conclusions:**

Previous admission for vaginal bleeding and change in CL are independent predictors of emergency CS in placenta previa. Women with change in CL more than 6 mm between the second and third trimester are at high risk of emergency CS in placenta previa. Single measurements of short CL at the second or third trimester do not seem to predict emergency CS.

## Introduction

Placenta previa is one of the most serious complications during pregnancy, because of possible emergency cesarean section (CS) if abrupt and massive hemorrhage occurs in the antepartum period. Massive bleeding can lead to serious maternal morbidity and even mortality, and emergency preterm delivery can contribute to neonatal morbidity or mortality. However, it is not easy to predict the risk of maternal bleeding and premature delivery in asymptomatic women. It is known that emergency CS due to uncontrollable bleeding is performed in 37% of patients with placenta previa, before the date of the elective CS [1]. Thus, maternal and neonatal morbidity would improve in patients with placenta previa, if the risk of bleeding during pregnancy could be predicted.

In theory, such complications (maternal hemorrhage, prematurity) could be expected more frequently in patients whose risk of preterm labor is increased [2]. Ultrasound measurement of cervical length (CL) has been shown to predict preterm delivery [3–5] and the date of delivery in asymptomatic women [6,7]. An inverse relationship between CL measured by transvaginal ultrasound and the risk of spontaneous preterm labor has been clearly demonstrated [8,9]. However, results regarding placenta previa are conflicting. Some studies reported an association between short CL and the risk of emergency CS in patients with placenta previa [2,10–12], but another study failed to demonstrate the correlation [1]. Furthermore, CL was measured only once in the third trimester in women at a wide range of gestational ages in previous reports. In addition to a single measurement of CL, a recent study focused on the relationship between the change in CL over time and preterm CS following massive hemorrhage in women with placenta previa [13]. However, no previous studies evaluated the clinical value of cervical changes in predicting adverse perinatal outcomes.

Therefore, the aim of this study was to assess the value of serial transvaginal CL measurements as predictors of emergency CS in placenta previa. We also sought to explore whether change in CL leads to a better prediction than single CL measurements in the second and third trimester.

## Methods

This was a retrospective cohort study in which pregnant women with placenta previa were recruited at Bucheon St. Mary’s Hospital, Kyonggido, Korea, between January 2010 and November 2014. This study was approved by the ethics committee of the Clinical Research Coordinating Center of the Catholic Medical Center (XC14RISI0095). The institutional review board waived the need for written informed consent from the participants for their information, because this study was retrospective study and data were analyzed anonymously.

Inclusion criteria were women who delivered neonates at our hospital and who were diagnosed as having placenta previa at delivery. We excluded patients with any of the following: low-lying placenta, multifetal gestation, preterm delivery without vaginal bleeding, premature rupture of membrane, history of conization, presence of cerclage, maternal disease or hypertensive disorder during pregnancy, and clinical chorioamnionitis. For participants, the medial charts were then reviewed.

Placenta previa was diagnosed by experienced obstetricians, based on transvaginal ultrasound showing the internal os covered by the placenta. Placenta previa was termed complete if the placenta completely covered the internal os, and incomplete if the placental edge partially covered or reached the margin of the internal os [14]. The primary placental location was described as anterior or posterior.

In our practice, we routinely assess serial CL measurements in women with placenta previa, from early gestation to delivery. All women in the study were offered serial measurement of CL by transvaginal ultrasound at 19 to 23 weeks (CL1), 24 to 28 weeks (CL2), 29 to 31 weeks (CL3), and 32 to 34 weeks (CL4). CL was measured by a straight line from the internal os to the external os, on a clear view of the cervical canal. At least three measurements were obtained and the shortest measurement was recorded. Transvaginal ultrasound was performed with Accuvix XQ and V10 (Samsung Medison Co., Ltd., Seoul, Korea) equipped with a 5 MHz transvaginal transducer.

Elective CS was planned between 37 and 38 completed weeks of gestation. If a patient visited due to vaginal bleeding before the planned CS, she was admitted for observation. Emergency CS was performed according to the clinical status of the patient. In a case of massive bleeding, emergency CS was performed. If vaginal bleeding stopped after admission, the patient was discharged until elective CS.

Maternal characteristics were compared between elective and emergency CS groups. Serial changes in CL during the second and third trimester of the two groups were also analyzed. The change in CL (expressed as mm) was defined as the difference between the initial CL (CL1) and follow-up measurement at the third trimester (CL4). A short CL was defined as a single measurement less than 25 mm. We used CL1 < 25 mm as a short CL at the second trimester (19–23 weeks), and CL4 < 25 mm at the third trimester (32–34 weeks). The relationship between change in CL and emergency CS for hemorrhage was examined.

Sample size estimates were based on the assumption that among women with placenta previa, the ratio of the emergent CS and elective CS groups would be 1:2. For the ratio of CL change, a sample size of 99 would be sufficient to identify an absolute increase of 26% between the groups, from 10% with elective CS group to 36% with emergent CS group, with 80% power.

All statistical analyses were performed using SPSS version 20.0 (SPSS Inc., Chicago, IL, USA). Demographics of women were compared using chi-square analysis or Fisher’s exact test for categorical variables, and the Student *t* test or Mann-Whitney *U* test for continuous variables. The significance level was defined as a p value less than 0.05. The odds ratio (OR) for emergency CS was calculated, using logistic regression analysis to control for possible confounders, which were maternal age, admission for bleeding, complete placenta previa, anterior placenta, change in CL, CL1 < 25 mm, CL4 < 25 mm, nulligravida, nullipara, prior CS, and prior preterm delivery. The adjusted OR and 95% confidence interval (CI) were analyzed. A receiver operating characteristic (ROC) curve was plotted to assess the value of change in CL for predicting women at high risk for emergency CS due to massive bleeding. The optimal cutoff point of CL was determined according to the highest sensitivity and specificity.

## Results

During the study period, 1401 deliveries were recorded in the hospital; of these, 143 (10.2%) were in women with placenta previa. A total of 50 women were excluded due to low lying placenta (n = 23), multifetal gestation (n = 3), preterm delivery without vaginal bleeding (n = 4), premature rupture of membrane (n = 5), history of conization (n = 2), presence of cerclage (n = 1), maternal disease or hypertensive disorder during pregnancy (n = 3), clinical chorioamnionitis (n = 1), and loss of follow-up (n = 8), which left 93 women for evaluation. Of these, emergency CS was performed in 31 (33.3%) women.

The clinical characteristics of patients are presented in Table 1, with a comparison between the emergency and elective CS groups. There were no significant differences between the two groups with regard to maternal age, percentage of nulligravida, prior preterm or CS, low Apgar score, type of placenta previa, and placental location. The emergency CS group was associated with a lower percentage of nullipara (25.8% versus 51.6%, p = 0.018), and more admissions for vaginal bleeding (90.3% versus 33.9%, p < 0.001), earlier gestational age at delivery (35 ± 3 versus 38 weeks ± 1, p < 0.001), and higher neonatal birth weight < 2000 g (24.8% versus 0%, p < 0.001) than the elective CS group.

**Table 1:** Clinical characteristics of the patients.

	Emergency CS group (n = 31)	Elective CS group (n = 62)	p value
**Maternal age (y)**	35.8 ± 4.0	35.4 ± 4.2	0.685
**Nulligravida**	6 (19.4)	19 (30.6)	0.247
**Nullipara**	8 (25.8)	32 (51.6)	0.018
**Prior preterm delivery**	3 (9.7)	2 (3.2)	0.193
**Prior cesarean delivery**	9 (29)	14 (22.6)	0.497
**Admission for bleeding**	28 (90.3)	21 (33.9)	< 0.001
**Gestational age at delivery (wk)**	35 ± 3	38 ± 1	< 0.001
**Birth weight (g) < 2000 g**	8 (24.8)	0 (0)	< 0.001
**AS 5 minute < 7**	9 (29)	8 (13.1)	0.063
**Type of placenta previa**			0.257
** Complete**	21 (67.7)	37 (59.7)	
** Incomplete**	10 (32.3)	15 (40.3)	
**Placental location**			0.151
** Anterior**	7 (22.6)	7 (11.3)	
** Posterior**	24 (77.4)	55 (88.7)	

CS, cesarean section; AS 5, Apgar score at 5 minute

Values are expressed as mean ± standard deviation or numbers (%).

**Fig 1:**
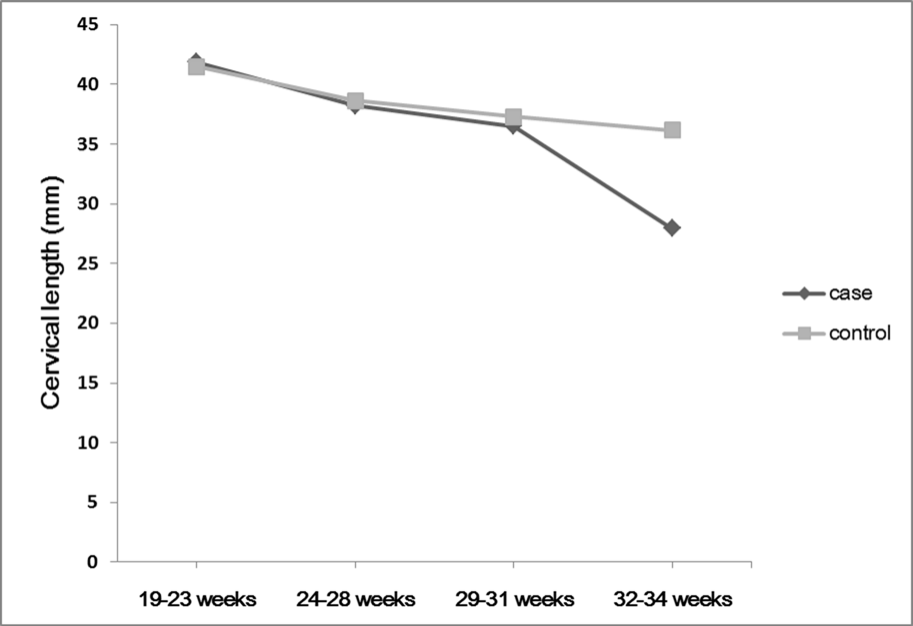
Serial changes in cervical length from 19 to 34 weeks comparing two groups. The black line represents the cervical length of women in the emergent cesarean delivery group, and the gray line represents that of the elective cesarean group.

Fig 1 shows the serial changes in cervical length from 19 to 34 weeks in the two groups. All 4 CL measurements were available for all participants. The mean CL of the emergency CS group at 19–23, 24–28, 29–31, and 32–34 weeks was 41.83, 38.21, 36.50, and 27.93 mm, respectively. The mean CL of the elective CS group was 41.46, 38.61, 37.27, and 36.15 mm, respectively. CL tended to decrease with advancing gestational age in each group. Until 29–31 weeks, CL showed no significant differences between the two groups, but after that, CL in the emergent CS group decreased abruptly, even though CL in the elective CS group continued to gradually decrease.

To determine whether the association between change in CL and risk of emergency CS is independent, or is the result of confounding effects, multivariable logistic regression analysis was used. Table 2 shows associations for risk factors for emergency CS in patients with placenta previa. In the univariate analysis, women in the emergency CS group were more likely to have admissions for vaginal bleeding (OR, 18.222; 95% CI, 4.958–66.973; p < 0.001), change in CL (OR, 3.386; 95% CI, 1.805–6.350, p < 0.001), CL4 < 25 mm (OR, 10.817; 95% CI, 2.739–42.723, p = 0.001), and less nullipara (OR, 0.326; 95% CI; 0.127–0.840; p = 0.020) than women in the control group. In the multivariate analysis that controlled for maternal age, admission for bleeding, complete placenta previa, anterior placenta, change in CL, CL1 < 25 mm, CL4 < 25 mm, nulligravida, nullipara, prior CS, prior preterm delivery, admission for vaginal bleeding and change in CL were still significantly associated with a higher risk of emergency CS with an odds ratio of 34.7 and 3.5, respectively. No significant difference was noted in the rate of short CL at the third trimester (CL4 < 25 mm) or nullipara.

**Table 2:** Unadjusted and adjusted odds ratio of risk factors for emergent cesarean delivery in placenta previa.

	Unadjusted OR			Adjusted OR^a^		
	OR	(95% CI)	p value	OR	(95% CI)	p value
**Maternal age**	1.022	0.920–1.136	0.682	0.905	0.761–1.077	0.261
**Admission for bleeding**	18.222	4.958–66.973	< 0.001	34.710	5.239–229.973	< 0.001
**Complete placenta previa**	1.419	0.572–3.518	0.450	0.419	0.094–1.824	0.243
**Anterior placenta**	2.292	0.724–7.253	0.158	2.795	0.532–14.679	0.225
**Change in CL**	3.386	1.805–6.350	< 0.001	3.522	1.210–10.253	0.021
**CL1 < 25 mm**	2.033	0.123–33.641	0.620	1.465	0.054–39.457	0.820
**CL2 < 25mm**	0	0	0.999	0	0	0.999
**CL3 < 25mm**	3.214	0.508–20.332	0.215	0.074	0–37.326	0.413
**CL4 < 25 mm**	10.817	2.739–42.723	0.001	3.757	0.357–39.553	0.270
**Nulligravida**	0.543	0.192–1.540	0.251	2.534	0.290–22.144	0.401
**Nullipara**	0.326	0.127–0.840	0.020	0.100	0.010–1.009	0.051
**Prior CS**	1.403	0.528–3.729	0.498	0.408	0.074–2.248	0.303
**Prior preterm**	3.214	0.508–20.332	0.215	1.939	0.023–163.362	0.770

OR, odds ratio; CI, confidence interval; CL, cervical length; CS, cesarean section

Values are expressed as odds ratio (95% confidence interval)

^a^Adjusted for maternal age, admission for bleeding, complete placenta previa, anterior placenta, change in CL, CL1 < 25 mm, CL4 < 25 mm, nulligravida, nullipara, prior CS, and prior preterm delivery

**Fig 2:**
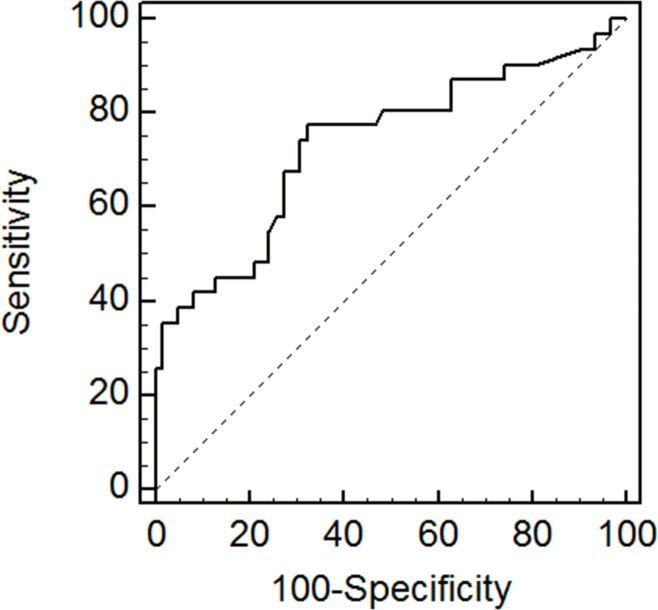
Receiver operating curve for change in cervical length in the prediction of emergency cesarean section in women with placenta previa.

The ROC curve for change in CL in the prediction of emergency CS for vaginal bleeding is shown in Fig 2. Based on the ROC curve at a cutoff point > 6.0 mm for change in CL, the sensitivity, specificity, positive predictive value (PPV), and negative predictive value (NPV) were 77.4, 67.74, 54.5, and 85.7%, respectively, for predicting cases at high risk for emergency CS, with area under the curve (AUC) 0.734 (p < 0.001).

## Discussion

Our study shows that a previous admission for vaginal bleeding and change in CL are independent predictors of emergency CS in placenta previa. Women with a change in CL greater than 6 mm between the second and third trimester are at high risk of emergency CS in placenta previa. However, it may not be recommended to use it as the only diagnostic clinical parameter, because its predictive value is moderate. Single measurements of short CL at the second or third trimester do not seem to predict emergency CS. This study might help to identify asymptomatic patients with placenta previa at high-risk of emergency CS.

Previous studies showed that CL can be a tool to predict hemorrhage in placenta previa. Ghi et al. reported that CL in the third trimester was significantly shorter in patients with placenta previa who underwent CS before 34 weeks, compared with patients who underwent elective CS [2]. Others also reported that a decrease in CL < 30 or 35 mm in any gestation was associated with increased risk of preterm CS due to massive hemorrhage [10,13]. However, these studies had limitations, in that CL was checked at various points of gestational age; single measurements may not be representative of CL, because some women have a short CL initially, and mean values reported in the literature at fixed points in pregnancy are quite variable [9,15,16]. For example, mean values for nulliparas vary from 34 to 41 mm at around 24 weeks, and from 23 to 30 mm at term [9,15,16]. Another study also argued that a single measurement of CL is not sufficient to predict the outcome [13]. A recent study showed that CL in women with placenta previa is a useful predictor of emergency CS due to bleeding or labor, and improved its utility by using CL *Z*-scores adjusted for gestational age [17].

Instead of a single measurement of CL, we used the change in CL over 2 measurements between the second and third trimester as a significant predictor of emergency CS in asymptomatic pregnancies. Another study of twin pregnancies also showed that one CL demonstrating a short cervix has predictive value for preterm birth, and serial CL measurements that demonstrate shortening can identify another high-risk group for spontaneous preterm birth, even in the absence of a short CL [18]. In placenta previa, a previous study performed serial measurements of CL every 2 weeks, but the change in CL was not taken into account as a predictor of emergency CS [13]. To our knowledge, this is the first study to evaluate change in CL as a predictor of adverse outcome in women with placenta previa.

We also showed serial changes in CL during the second and third trimester in women with placenta previa. Serial change in CL is characterized by three patterns in normal patients: unchanged prior to delivery, progressive shortening within 2 weeks prior to delivery, and steady shortening starting before 2 weeks prior to delivery [19]. In this study, change in CL in the emergency CS group showed that CL shortened abruptly. With this result, we can assume that the rate of change from second to third trimester, rather than a single measurement during a gestation, can help to predict emergency CS. Other studies also showed that cervical shortening was a significant independent predictor of preterm delivery [20,21].

The mechanism of hemorrhage in placenta previa is not well known, but cervical effacement and labor are thought to be the cause of bleeding.The uterine cervix undergoes a series of changes during pregnancy, resulting in gradual reduction of mechanical strength, and eventual effacement and dilatation [22,23], resulting in placental vessel tearing [16,22,24]. Inability of the lower uterine segment myometrium to constrict the torn vessels [25] can cause massive vaginal bleeding, necessitating emergency CS. This mechanism can justify use of CL shortening as a predictor of bleeding, because it represents cervical effacement and dilation, and is already known to be associated with preterm labor. For this reason, hemorrhage in placenta previa typically occurs in the third trimester, as the lower uterine segment becomes more defined, and the internal os dilates [11].

Our study also showed that a previous admission for vaginal bleeding was associated with increased risk of emergency CS, because of recurrent massive vaginal bleeding. A previous study showed that patients with episodes of antenatal bleeding had more emergency CS than patients without bleeding [1]. For patients discharged after observation for bleeding, a warning of the possibility of emergent CS should be given. Hospital admission for vaginal bleeding was a stronger predictor parameter than change in CL, which supports the hypothesis that the highest value of change in CL would be found in asymptomatic patients without vaginal bleeding.

There are some limitations to our study. First, this was a retrospective review, which precludes control for additional risk factors for emergency CS. Second, our study only recruited a small number of women.

Despite these limitations, our study has several strengths. Ours is the first to use cervical change to predict the outcome of placenta previa. In addition, we compared not only single CL measurements at the second and third trimester, but also compared change in CL concurrently, in order to assess independent predictors of emergency CS. In addition, we described serial changes in CL with advancing gestational age in placenta previa.

In cases of CL shortening without symptoms, we can educate patients earlier, to prevent massive bleeding. Prenatal assessment of factors associated with a high risk of emergency CS would improve the preparation and management of such patients, and can help decide whether to prepare for blood in advance, and when to schedule CS. For asymptomatic women with placenta previa, our study suggests that a change in CL greater than 6 mm from the second to third trimester gives useful clinical information on the likelihood of emergency CS.
